# Stochastic virtual population in type 1 diabetes

**DOI:** 10.1371/journal.pone.0341034

**Published:** 2026-02-06

**Authors:** Mate Siket, Levente Kovacs, Gyorgy Eigner

**Affiliations:** 1 Biomatics and Applied Artificial Intelligence Institute, John von Neumann Faculty of Informatics, Obuda University, Budapest, Hungary; 2 Physiological Controls Research Center, Obuda University, Budapest, Hungary; 3 MedTech Innovation and Education Center, Obuda University, Budapest, Hungary; University of Milano Bicocca, ITALY

## Abstract

Accurate, reliable, and efficient estimation of blood glucose dynamics from real-world data is challenging due to the time-varying nature, high uncertainty, and nonlinear interplay of complex processes. In this study, we propose and investigate a stochastic representation of a virtual population by fitting a hierarchical Bayesian model. In total, we use 500 24h-long sequences, 50 from each of the 10 patients with type 1 diabetes on multiple daily injection therapy. We model uncertainty on multiple levels, in physiology and in self-reported events, and take into account intra- and interday variability, and the effect of physical activity as well. The root-mean-square error between the glucose measurements and the mean of the posterior predictive distribution using the fitted low-rank multivariate normal guide is 12.44 mg/dL. We show that the posterior distributions can be used to simulate realistic intra-, and interday variability in terms of the investigated patient cohort.

## Introduction

Type 1 diabetes mellitus (T1DM) is characterized by the complete absence of insulin production. People with T1DM administer exogenous insulin to keep their blood glucose level in a healthy range. Insulin can be intermittently injected and it is called multiple daily injection (MDI) therapy, or administered in microboluses by an insulin pump. By observing the input-output dynamics of the blood glucose level a digital representation of the patient, a model can be developed. In general, these models can be used to simulate how the blood glucose changes with respect to the inputs. The simulations are valuable in optimizing the therapy of the patient, developing/testing algorithms that determine the amount of insulin, reasoning about the physiology of the patient, or for educational purposes.

The development of models and the estimation of the parameters of those models have considerable challenges. Physiology is not time-invariant, it is a highly complex system with interacting hormones affected by external stimuli. Patients exhibit intrapatient variability through diurnal, seasonal variation, but also there are lot of factors that are not known or difficult to model, such as mood, physical activity, stress. There is huge uncertainty even in the self-reported events such as in the carbohydrate content of the meal. Glycemic index, macronutritional composition, and individual factors may also affect how glucose appears in the plasma. In this highly uncertain environment, it is difficult to come up with a digital representation of the patient, especially so if the blood glucose dynamics is estimated based on a short sequence of data, such as a couple of hours. Although it is difficult to model and take into account different variabilities and uncertainties, it is crucial in the development of control algorithms [1,2], and in general in simulations to provide a more realistic testbed.

A recent review [3] on digital twins in T1DM summarized different methods to represent a patient’s blood glucose dynamics. Hughes et al. [4] developed a method to replay different inputs; the method uses maximum likelihood estimation of the parameters based on a short sequence of data. Variability, uncertainties, and unmodeled phenomena are absorbed in a residual “net effect” signal. Cappon et al. developed [5,6] an open-source tool for creating digital twins, and replaying different inputs. It uses Bayesian estimation to determine the parameters of the model, but relies on a short sequence of data. Haidar et al. [7] proposed a full Bayesian approach as well; they coined their method as “stochastic e-cloning”. Yue Ruan et al. [8] proposed a hierarchical Bayesian model to represent intraday variability based on multiple days of data, which most closely aligns with our work without taking into account diurnal variability, physical activity, and uncertainties in reported events. In a recent work, Aiello et al. [9] also investigated variabilities using a hierarchical structure.

To represent the uncertainty in various aspects of the blood glucose dynamics, we propose a stochastic representation of the patients using multilevel Bayesian modeling. Fitting the complex posterior distribution with a high number of latent variables when the likelihood calculation involves the numerical integration of a differential equation is a challenging task. Thus, we utilize stochastic variational inference (SVI) to estimate cohort and patient-level distributions of 10 patients, each with 50 sequences, each sequence containing a 24-hour-long duration of data. The posteriors can be used to simulate the variability during and between days. The model uses insulin injections, meal intakes, physical activities, and heart rate as inputs. The model is fitted using SVI on data from 10 participants on MDI therapy under free-living conditions.

The paper intentionally presents the methods in a concise manner since all the results, implementation, along with the dataset is open-source available at [10].

## Materials and methods

### Dataset

Data were collected from November 10, 2023, to September 16, 2024 in the study titled “Exploratory observational study to investigate the relationship between type 1 diabetes and physical activity”. The study was carried out in full compliance with ethical standards and guidelines for research involving human participants. The study was approved by the Scientific and Research Ethics Committee of the Health Science Council (Egészségügyi Tudományos Tanács, Tudományos és Kutatásetikai Bizottság) under the number of IV/173-6/2022/EKU. All participants provided informed written consent prior to participation, the study adhered to the principles outlined in the Declaration of Helsinki, and data was stored in de-identified format. 10 participants (HbA1c of 7.1 ± 0.6%, age of 35 ± 7.1 years, and body weight of 73 ± 9.4 kg) with type 1 diabetes wore a CGM device (Medtronic Guardian 3), activity tracker, and reported their daily insulin injections, meal intakes, and physical activities in a smartphone application over several weeks [11]. All of the participants were on MDI therapy, and asked to follow their regular routine, thus the data collection was under free-living conditions. Meal intakes could be selected from a large database of the Diabtrend smartphone application with predefined macronutritional composition or they could manually entry them. Physical activities were self-reported in the application, heart rate was measured by Xiaomi Miband 6 activity tracker.

24h-hour-long sequences were extracted with strides minimum of 5 hours; each sequence represents 1 patient sample. The samples were filtered to have: 1) less than 12 minutes of gap in CGM, 2) less than 45 mg/dL discontinuity in CGM, 3) at least 2 meal intakes, 4) at least 1 insulin injection, 5) at least 30% of the time heart rate values. After the collection of the day-long samples, 50 of them were used from all 10 patients, resulting in a total of 500 24h-long samples. Long-acting insulin injections were assumed to have a constant effect in the next 24 hours. In total, the dataset contains 1349 reported meal intake, 931 fast-acting injection, and 164 physical activity events. Table 1 summarizes the key information of the dataset.

**Table 1:** Summary of the diabetes dataset.

Demographics	Description
Number of Participants	10
Condition	Type 1 Diabetes
Age	35 ± 7.1 years
Body Weight	73 ± 9.4 kg
HbA1c	7.1 ± 0.6%
Therapy	MDI (Multiple Daily Injections)
Data sources, variables	**Description**
CGM	Medtronic Guardian 3
Activity Tracker	Xiaomi MiBand 6 (for heart rate)
Data Logging Application	Diabtrend (for insulin, meals, activity)
Meal Data	From database or manual entry
Insulin Data	Fast-acting and basal injections
Physical Activity	Self-reported by user
Heart Rate	Measured by activity tracker
Statistics	**Description**
Sample Length	24 hours
Total Number of Samples	500 (10 patients, 50 samples/patient)
Total Meal Intakes	1349
Total Fast-acting Injections	931
Total Physical Activities	164

### Probabilistic model

**Fig 1:**
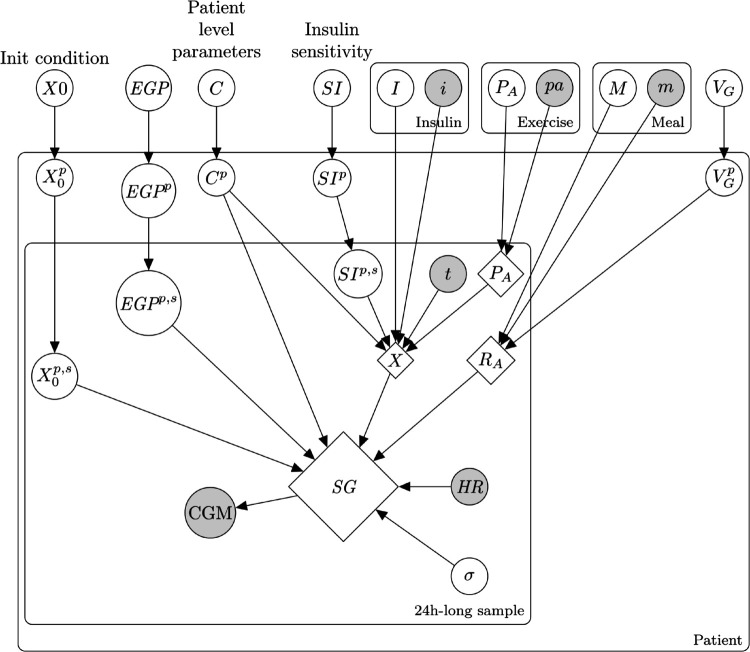
Graphical model of the probabilistic model. Observed variables are the *i* insulin injections, *pa* physical activity events, *m* meal intakes, *t* time, CGM glucose measurements, SG subcutaneous glucose concentration, and HR heart rate measurements. X is the effect of the insulin, *R*_*A*_ is the rate of glucose absorption from the carbohydrate intake, *P*_*A*_ is the effect of physical activity on the insulin sensitivity, $V_{G}$ is the glucose distribution volume. Parameters vary in terms of number of levels of estimation, but there are four distinct levels: cohort, patient, sample (corresponding to a 24h-long window), and event (such as physical activity, meal intake, or insulin injection). Table 2 details the latent variables and their estimation method. The deterministic submodels (in diamonds) are differential equations solved by numerical integration or through their analytical impulse response.

To represent uncertainty and variability in physiology and in patient behavior, we use a probabilistic model. More specifically, we use a hierarchical Bayesian model which permits us to represent uncertainty on multiple levels and to pool information from multiple samples. In addition, by drawing samples from the posterior distributions, new patients, diurnal, and day-to-day variability can be generated. Our prior beliefs about the physiology, uncertainties, and variabilities are embedded in the deterministic submodels and in the prior distributions. Fig 1 shows a graphical representation of the proposed probabilistic model. Each node aggregates multiple random variables as given in Table 2. Variables differ in the number of levels on which they are estimated. For instance, the initial condition is estimated on the cohort, patient, and sample level as well, while the glucose distribution volume is only estimated on the cohort and patient level. For some of the key variables, the variance is also estimated. Such key variables are insulin sensitivity and endogenous glucose production which account for most of the variability observed in the patients glucose dynamics between different 24h-long samples.

**Table 2:** Summary for the latent and observed variables of the model.

Node	Variable	Level	Description
*M*	*T*_*D*_	Meal	Meal start offset. Uncertainty in the self-reported time, delay in absorption
*M*	$\tau_{D1}$	Patient	Meal absorption time constant 1
*M*	$\tau_{D2}$	Patient, Meal	Meal absorption time constant 2.
*M*	*M*_*A*_	Meal	Carb coefficient. Uncertainty in the self-reported carbohydrate content.
$V_{G}$	$V_{G}$	Cohort, Patient	Glucose distribution volume.
*m*	Self-reported carb content	Meal (Observed)	
*I*	*T*_*I*_	Bolus	Bolus start offset. Uncertainty in the self-reported time, delay in absorption.
*I*	$\tau_{I1}$	Patient, Bolus	Bolus time constant 1. Captures variability in absoprtion with respect to injection site.
*I*	*I*_*A*_	Bolus	Bolus coefficient. Uncertainty in the self-reported bolus injection.
*i*	Self-reported bolus	Meal (Observed)	Short-acting insulin
*X*_0_	*X*_0_, *S*_0_, *I*_*p*0_	Cohort, Patient, Sample	Initial state of the model, glucose values are based on CGM at 0 time instance.
*EGP*	*EGP*	Sample	Endogenous glucose production: $TN(EGP_{i}+EGP_{m}\;\cdot \;SI_{E},\sigma )$
*EGP*	$EGP_{i}$	Cohort, Patient	Endogenous glucose production at zero insulin sensitivity
*EGP*	$EGP_{m}$	Patient	Slope between *SI* and *EGP*
*C*	*GEZI*	Cohort, Patient	Glucose effectiveness at zero insulin *GEZI*
*C*	$\tau_{I2,3,4}$	Cohort, Patient	Insulin absorption time constants
*C*	$\tau_{G}$	Cohort, Patient	Delay due to subcutaneous measuring site of the glucose.
*C*	*H*	Cohort, Patient	Effect of increased insulin sensitivity in hypoglycemia.
*C*	*G*_*th*_	Cohort, Patient	Threshold glucose level for the increased insulin sensitivity in hypoglycemia.
*C*	*SE*	Cohort, Patient	Effect of elevated metabolic rate parameterized by excess heart rate over the resting value.
*C*	$\tau_{E}$	Cohort, Patient	Time constant of the metabolic rate.
*SI*	*SI*_*E*_	Cohort, Patient, Sample	Expected value of the insulin sensitivity without physical activity and intraday variation.
*SI*	*SI*_*A*_	Cohort, Patient, Sample	Amplitude of the intraday sinusoidal insulin sensitivity variation.
*SI*	$SI_{\Phi }$	Cohort, Patient, Sample	Phase of the intraday sinusoidal insulin sensitivity variation.
*P*_*A*_	*T*_*P*_	Exercise	Uncertainty in the self-reported time
*P*_*A*_	$\tau_{P,long}$, $\tau_{P,short}$	Patient, Exercise	Time constant of the short and long effect of physical activity.
*P*_*A*_	*P*_*long*_, *P*_*short*_	Exercise	Gain of the short and long effect of physical activity.
*p*	Self-reported physical activity	Exercise (Observed)	
*HR*	Measured heart rate	Measurement (Observed)	
*CGM*	Measured blood glucose	Measurement (Observed)	
*SG*	Subcutaneous glucose level	Variable (Observed)	Model output
*σ*	CGM measurement uncertainty	Cohort, Patient, Sample	

The deterministic submodel builds on previous works in modeling the absorption of insulin and glucose, and their effects on blood glucose [5,12,13]; the renal excretion is borrowed from the Hovorka model [14]. Heart rate as the driver of elevated metabolic rate is a greatly simplified version of the submodel described in [15,16].

Several different models were developed to take into account the effect of physical activity. Deichmann et al. [17] modeled the effect on insulin-independent glucose uptake, production and prolonged rise in insulin sensitivity with accelerometry data as the driving variable. Hobbs et al. [18] differentiated fast-acting, high-intensity, and slow-acting effects of physical activity. Mosquera-Lopez et al. [19] analyzed the risk of hypoglycemia following a physical activity and found that there is an increased risk both shortly after the exercise and again several hours later. Dalla man et al. [20] also take into account short- and long-term effects of physical activity using heart rate as the driving variable. Despite some common threads in physiology-based models of physical activity, there is no widely accepted and proven model due to the heterogenity (in modality, intensity) of the activities and also due to the variability in the physiological responses. We aimed to reflect the short and prolonged effects of physical activity, but instead of using a complex physiology-based model with multiple assumptions, we wanted to make little assumptions. We model the effect of self-reported physical activity as a combination of short- and long-lasting perturbation in insulin sensitivity with exponential decay. Perturbation of insulin sensitivity due to physical activity was also modeled by Alkhateeb et al. [21].

The diurnal variability in insulin sensitivity was investigated in multiple studies [22–24]. In this study, we model it as a sinusoid which can have varying amplitude and phase between different 24h-long samples; this assumption was also made in previous works [25–28].

The deterministic submodel is summarized as follows:


$$SG=G\overset{\;\;}{\underset{\tau_{G}\;}{\to }}SG,\;\dot{G}=-(GEZI+h\;\cdot \;X)G+EGP\;-SE\;\cdot \;E+R_{A}-F_{R},\;h=\left\{ \begin{array}{c} H,\;\text{if}\;G<G_{\mathrm{th}},\\ 0,\text{ otherwise}. \end{array}\right.\;F_{R}=\left\{ \begin{array}{c} 0.003(G-162),\;\text{if}\;G>162\;\mathrm{mg}/\mathrm{dL},\\ 0,\text{ otherwise}. \end{array}\right.\;SI={SI}_{E}\;\cdot \;(1+P_{A})\;\cdot \;(1+{SI}_{A}\;\cdot \;\sin\left(2\pi (t+{SI}_{\phi })\right)),\;X=\text{insulin}\overset{\;\;}{\underset{\tau_{I1}\;}{\to }}S_{1}\overset{\;\;}{\underset{\tau_{I2}\;}{\to }}S_{2}\overset{\;\;}{\underset{\tau_{I3}\;}{\to }}I_{p}\overset{SI\;}{\underset{\tau_{I4}\;}{\to }}X,\;R_{A}=\text{meal}\overset{V_{G}\;}{\underset{\tau_{D1}\;}{\to }}D_{1}\overset{\;\;}{\underset{\tau_{D2}\;}{\to }}D_{2}\overset{\;\;}{\underset{\tau_{D2}\;}{\to }}R_{A},\;P_{A}=\text{exercise}\overset{P_{long},P_{short}\;}{\underset{\tau_{P,long},\tau_{P,short}\;}{\to }}P_{A}\;E=\text{HR}\overset{\;\;}{\underset{\tau_{E}\;}{\to }}E_{1}\overset{\;\;}{\underset{\tau_{E}\;}{\to }}E,$$

where *SG* is the subcutaneous glucose concentration, *G* is the blood glucose concentration, *h* is the coefficient for insulin sensitivity in hypoglycemia, *F*_*R*_ is the renal excretion, *SI* is the varying insulin sensitivity, *X* is the effect of insulin, *R*_*A*_ is the rate of appearance of glucose from meal intakes, *P*_*A*_ is the effect of physical activity on insulin sensitivity, *E* is the energy expenditure with respect to elevated heart rate.

Self-reported events such as meal intakes, fast-acting bolus injections, and physical activities were taken into account through their impulse response. Meal intakes directly affect the blood glucose through a third-order system parameterized by a mixture of meal-level and patient-level parameters. For instance, the glucose distribution volume is on the patient level, but some of the time constants and uncertainty in the carbohydrate report are estimated on the meal level. Physical activity is assumed to be first-order processes with short and long lasting effects; both of them alter the insulin sensitivity.

### Prior predictive check

We carried out prior predictive checks, and adjusted the priors so that the intervals of the parameters represent physiologically relevant ranges, reasonable uncertainty in the self-reported values, and the prior predictive distributions span the physiological blood glucose range. The type of the priors are listed in Table 3.

**Table 3:** Prior distributions.

Prior distribution	Variables
Truncated normal	Variables in node: *M*, $V_{G}$, *EGP*, *C*, *SI*, *P*_*A*_
Half-normal	Variance of $X_{0}^{p}$, $\tau_{I1}^{p}$
Exponential	Variance of *EGP*^*p*^, $SI_{E}^{p}$, $SI_{A}^{p}$, $SI_{\phi }^{p}$, $\tau_{D1}^{p}$, $\tau_{P,long}^{p}$
Uniform	$SI_{\Phi }^{c}$
Log-normal	*σ*

Superscript *p* denotes the patient level, *c* the cohort level.

**Fig 2:**
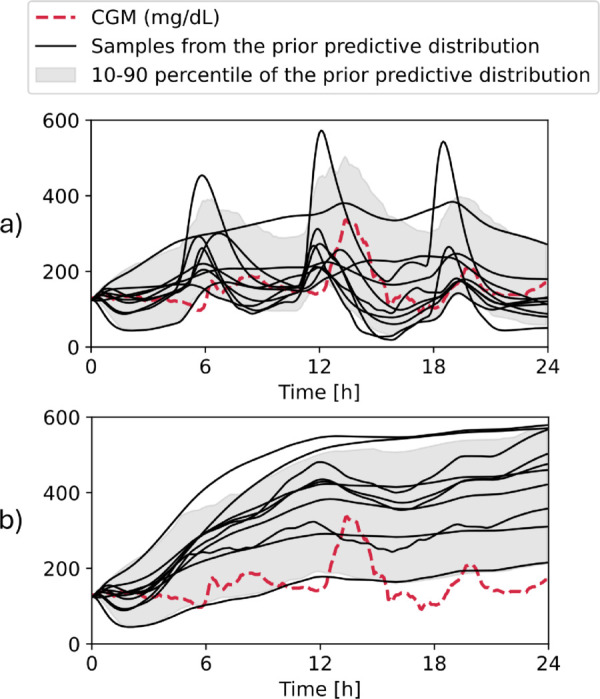
24h-long sample from the dataset (CGM, meal intake, insulin, physical activity) for prior predictive checks. Colored lines are random samples from the prior predictive distribution. a) The prior predictive distribution spans the 0-400 mg/dL physiologically relevant space. b) Prior predictive distribution when conditioning on zero insulin and meal intake. Glucose in such scenarios is expected to rise to approximately 400 mg/dL where ketones are dominantly produced, and renal excretion is also active.

Fig 2 illustrates such two checks; one is given an exemplary sequence of insulin, meal, and physical activity, the other assuming zero insulin.

### Model fitting

The investigated problem is complex due to the high number of latent variables, multilevel nature, and computationally expensive numerical integration. We utilized SVI in Numpyro version 0.18 [29] to fit the model; the method scales well with the high number of latent variables and samples. Also, Numpyro utilizes that the probabilistic model is implemented in JAX, end-to-end differentiable, just-in-time compiled, and vectorized. We used low-rank multivariate normal guide which keeps some of the covariance between the latent variables without exploding the problem into the unfeasible territory. The total fitting process using 15 samples for the approximation of the evidence lower bound, with 80,000 iterations, takes 2h:25min on a Google TPU v6e-1.

## Results

### Posterior predictive distribution

**Fig 3:**
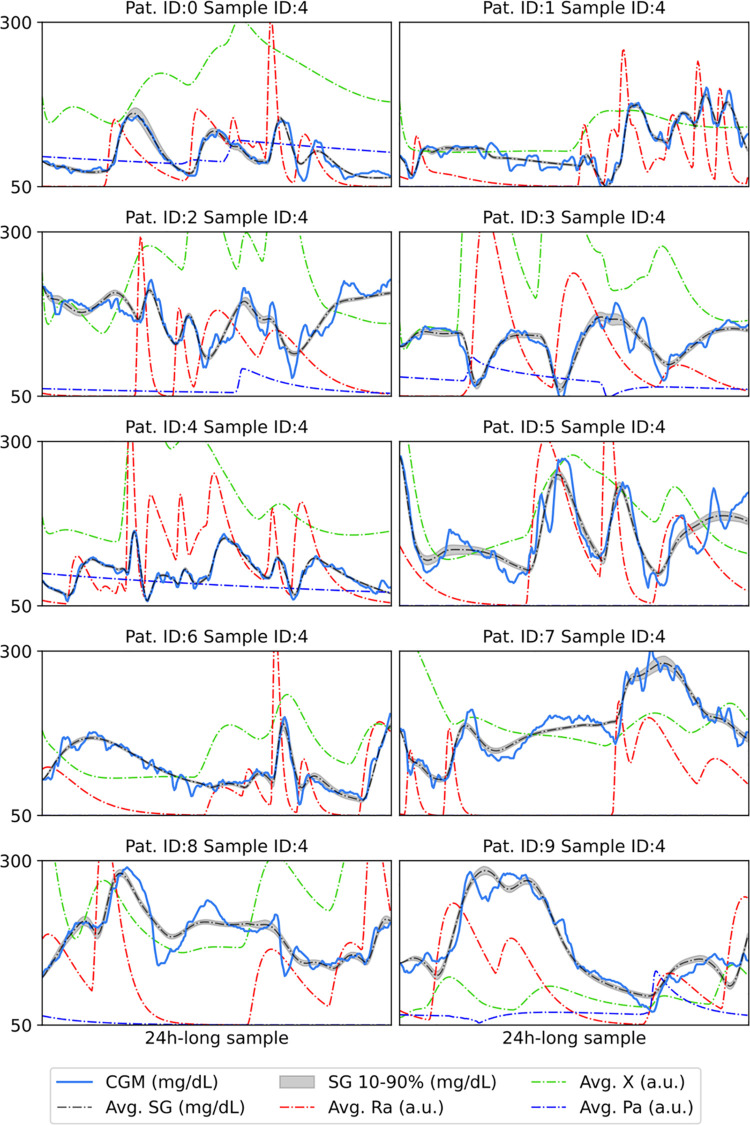
Exemplary fitted posterior predictive glucose trajectories for all the patients. Solid blue line shows the CGM measurements, black dashed line the posterior mean of the subcutaneous glucose, blue dashed line the coefficient of insulin sensitivity driven by physical activity, red line shows the rate of glucose appearance, green line shows the total effect of insulin.

Table 4 summarizes the root-mean-square error (RMSE) between the mean of the posterior predictive distribution and the CGM measurements. In the case of Patient 1 and 5, 6 mg/dL mean RMSE is achieved, while Patient 6 has the largest residual error with an average of 20.68 mg/dL. Fig 3 shows posterior predictive glucose trajectories fitted to exemplary samples for all the patients. Both the numerical RMSE values and the trajectories show a good fit with an overall average of 12.44 mg/dL RMSE.

**Table 4:** Root-mean-square error (mg/dL) between the CGM measurements and the mean of the posterior predictive distribution.

Patient ID	RMSE	(95% CI)
1	5.88	(4.93, 6.84)
2	7.58	(6.85, 8.30)
3	14.35	(9.90, 18.80)
4	15.94	(13.75, 18.13)
5	6.03	(5.59, 6.47)
6	20.68	(15.86, 25.49)
7	7.45	(6.64, 8.26)
8	13.69	(12.07, 15.30)
9	15.47	(14.17, 16.77)
10	17.37	(15.03, 19.71)

### Posteriors for the model parameters

**Fig 4:**
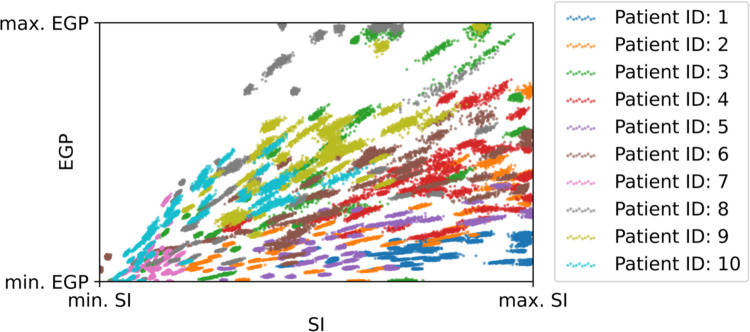
Samples from the posterior distributions of EGP and SI. One concentrated region belongs to a 24h-long patient sample.

In preliminary tests, we observed correlation between the sample-level posterior values of EGP and SI for some of the patients. Thus, we estimate a linear association between these two variables. Indeed, in Fig 4 with the exception of Patient 0 and 6 the posterior values of EGP and SI are visibly correlated. The correlation has to be estimated so that the variance in the observed glucose is not spuriously increased when generating new samples for the patients. This association between EGP and SI was also observed in [14] and was modeled similarly. Large variability can be observed between patients, but also between different 24h-long samples for a given patient.

Consistent increase in insulin sensitivity was not observed given the data, since for all the patients the coefficient *H* converged to the zero lower bound. Delay caused by the subcutaneous measurement site was estimated between 5 and 30 minutes; on the cohort level, a time constant of 20 min (95% CI: 17 to 23) was estimated with 2 patients converging to the lower and upper bounds. Cohort level $EGP_{i}$ was estimated to be 0.54 mg/dL/min (95% CI: 0.33 to 0.89), while *SI* $4.4\;\cdot \;10^{-4}$ mL/μU (95% CI $3.5\;\cdot \;10^{-4}$ to $5.4\;\cdot \;10^{-4}$). *GEZI* values of 7 participants converged to the lower bound (10^−3^ 1/min). Insulin absorption time constants ($\tau_{I2-4}$) on the cohort level were estimated to be 67 min (95% CI: 57 to 74), 67 min (95% CI: 57 to 74), 24 min (95% CI: 21 to 35), respectively. Insulin absorption on the cohort level in the first compartment $\tau_{I1}$ was 16 min (95% CI: 8 to 28). Carbohydrate absorption in the first compartment converged to the upper bound of 10 min. Measurement noise *σ* was assumed to be normally distributed and varying on the 24h-long sample level; the overall posterior mean was 13.5 mg/dL (CI: 5.1 to 31.3).

Intraday variation in insulin sensitivity was modeled on three levels (cohort, patient, and sample). It was constrained so that it could explain a maximum of 50% in variation of the insulin sensitivity. Table 5 summarizes the means and credible intervals on the patient level for the expected value of insulin sensitivity, endogenous glucose production, and maximum of the intraday varying insulin sensitivity. For most patients, the largest insulin sensitivity occurred in the dinner period followed by lunch, and with the least amount of occurrences during breakfast.

**Table 5:** Posterior means and 95% credible intervals for the parameters which affect interday variability.

Pat. ID	$SI_{E}$ ($10^{-4}\;\cdot \;mL/\mu U$)	*EGP* (mg/dL/min)	$\arg\max_{t}$ *SI* B, L, D (%)
1	6.75 (6.22, 7.19)	0.84 (0.77, 0.92)	24, 36, 40
2	5.52 (4.84, 6.07)	1.34 (1.18, 1.48)	18, 40, 41
3	4.43 (3.93, 4.86)	2.09 (1.87, 2.34)	22, 25, 53
4	6.69 (6.06, 7.20)	2.29 (2.10, 2.50)	24, 36, 40
5	4.79 (4.48, 5.08)	0.99 (0.90, 1.10)	21, 28, 51
6	5.35 (4.72, 5.92)	2.08 (1.88, 2.29)	20, 36, 44
7	1.28 (1.24, 1.34)	0.64 (0.58, 0.73)	31, 24, 45
8	3.34 (3.00, 3.67)	2.26 (2.02, 2.46)	24, 25, 50
9	4.15 (3.89, 4.39)	2.57 (2.41, 2.72)	19, 35, 46
10	2.09 (1.87, 2.29)	1.24 (1.12, 1.36)	27, 31, 42

Patterns of intraday variability with notations as B: Breakfast (2-10 h), L: Lunch (10-18 h), and D: Dinner (18-2 h).

**Fig 5:**
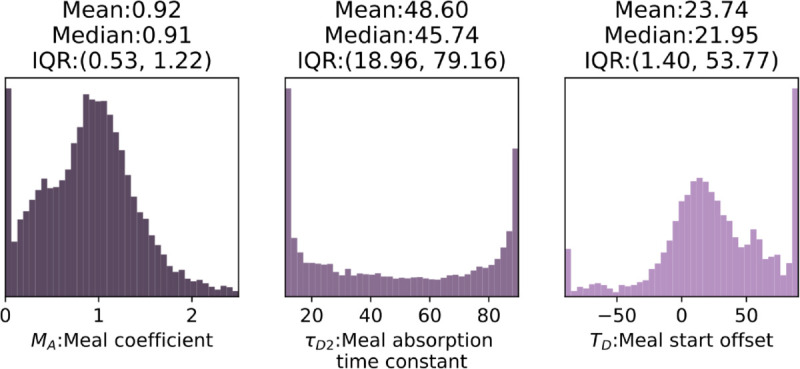
Histograms of the aggregated posterior samples of the variables related to the self-reported meals. The meal coefficients here are represented according to the 0.8-1.0 range of glucose bioavailability; the model was fitted assuming a bioavailability of 0.8.

A start offset, time constant, and a meal coefficient were estimated for each meal intake. Fig 5 represents aggregated samples for these variables given the model and the data. For 6% of the meal reports no effect was observed on the blood glucose. Both the mean and median lie close to one which imply no heavy bias in the self-reported carbohydrate content, but large variability is present. The model was allowed to account for a large uncertainty in the carbohydrate content with a standard deviation of 20%. Meal absorption time constants vary uniformly between the lower and upper bounds which shows large variability in the peak effect between different meals. Meal start offset can account for uncertainty in the time and duration of the meal consumption, and delay of appearance. The cumulative effect shows a mean delay of 22 min from the time of the report.

We carried out post-hoc analysis to investigate how macronutritional composition affects the meal-related parameters; the results are summarized in Table 6. Carbohydrate and fat content found to be positively (with a coefficient of 0.1 and 0.18 min/g) and significantly affecting the absorption time. However, the coefficient is small, 50 grams of extra carbohydrate would lead to a 5 min increase according to the model. We found no evidence on the effect of protein content on meal absorption time given the data and the model. Glucose level at the time of the reported meal was found to be negatively affecting the time constant with P = 0.03. Meal start offset was found to be positively affected by the carbohydrate content with 0.2 min/g (P = 0.002); fat and protein showed no evidence for affecting the offset. Glucose level at the self-reported time was negatively affecting the offset with –0.16 min/(mg/dL). Given the data and the model, there was evidence for the carbohydrate content negatively affecting the meal coefficient with –0.007 1/g, and for the fat content positively affecting with 0.004 1/g.

**Table 6:** Post-hoc analysis of the meal-related parameters with respect to macronutritional composition and glucose level at the self-reported time.

$\tau_{D2}$: Meal absorption time constant				
Variable	Unit	Coeff.	P>|t|	(0.025, 0.975)
Intercept	min	48.5	0.000	(45.0, 52.0)
Carbs	min/g	0.10	0.014	(0.021, 0.185)
Fat	min/g	0.18	0.047	(0.002, 0.349)
Protein	min/g	–0.01	0.927	(–0.177, 0.161)
ΔCGM	min/(mg/dL)	–0.04	0.027	(–0.069, –0.004)
**$T_{D}$: Meal start offset**				
Variable	Unit	Coeff.	P>|t|	(0.025, 0.975)
Intercept	min	35.91	0.000	(30.648, 41.164)
Carbs	min/g	0.20	0.002	(0.072, 0.317)
Fat	min/g	–0.17	0.210	(–0.424, 0.093)
Protein	min/g	–0.02	0.910	(–0.266, 0.237)
ΔCGM	min/(mg/dL)	–0.16	0.000	(–0.205, –0.109)
**$M_{A}$: Meal coefficient**				
Variable	Unit	Coeff.	P>|t|	(0.025, 0.975)
Intercept	1	1.010	0.000	(0.945, 1.075)
Carbs	1/g	–0.007	0.000	(–0.009, –0.006)
Fat	1/g	0.004	0.029	(0.000, 0.007)
Protein	1/g	–0.0003	0.830	(–0.003, 0.003)
ΔCGM	1/(mg/dL)	0.0003	0.345	(–0.000, 0.001)

ΔCGM: Measured glucose level - 40 mg/dL at the self-reported time.

**Fig 6:**
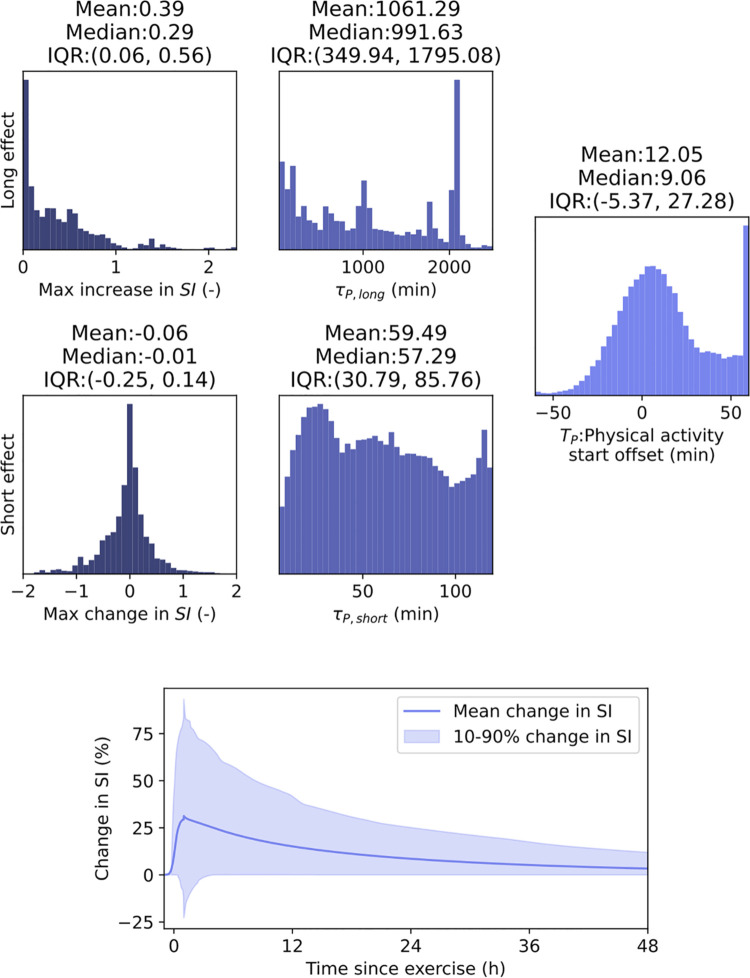
Histograms of the aggregated posterior samples of the variables related to the self-reported physical activities. The gain and the time constant differ between short and long effect, while the start offset was assumed to be the same. The lower subplot depicts aggregate effect of the posteriors.

For each self-reported physical activity a gain, time constant and start offset was estimated. The gains determine the maximum change in insulin sensitivity; long-term effect was assumed to only increase insulin sensitivity, while for the short-term effect no such assumption was made. It is important to note that in the 1-3 hours after physical activity both effects are present and they are superposed. After 2 hours, the short-term effect is assumed to taper off and only the long-term effect mediates the insulin sensitivity. Fig 6 collects the histograms of the posterior samples aggregated over all the events. For the long-term effect, the mean was a maximum increase of 40% in insulin sensitivity, but with large variability between different events. The duration of the effect also varies greatly with a mean half-time of 12 hours, but it can also extend into the following day. For the superposed short-term effect, no clear direction of effect was observed, with the time constant varying almost uniformly between 5 and 120 minutes. The start offset was estimated to be 10 min on average from the self-reported time. In posterior predictive checks, the exclusion of the effect of physical activity leads to an increase from a mean glucose of 144 to 162 mg/dL calculated on the whole cohort.

### Validation

**Fig 7:**
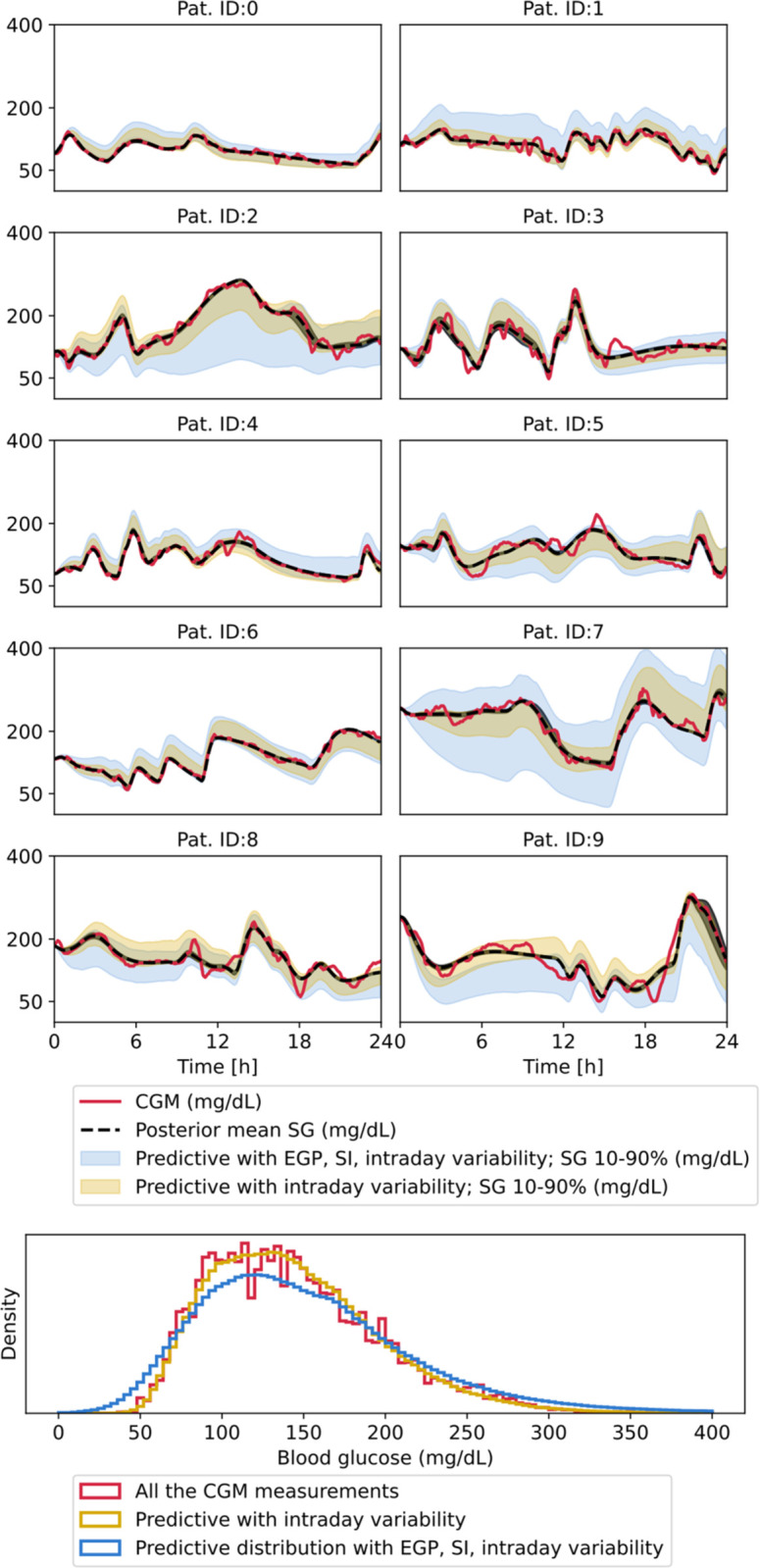
Posterior predictive checks with generating new EGP, SI, and intraday variability. The shaded area represent the 10-90 percentile of the resulting distribution. The black dashed line is the mean of the fitted posterior predictive distribution. The histogram compares the predictive distribution with different levels of uncertainty to the real-world CGM distribution. Yellow predictive intervals represent the uncertainty band caused by variability in the posterior parameters and intraday variation. The blue uncertainty band, in addition to intraday variability, also captures interday variability, which further inflates the bands.

We carried out posterior predictive checks by evaluating the capability of the fitted multivariate guide in generating physiologically plausible glucose trajectories when sampling EGP, SI, and intraday variability, keeping the effect of the meal, insulin, and physical activity the same. Fig 7 illustrates the distribution of the new samples for all the patients given a random 24h-long sequence. It can be observed that for both the intraday variation and combined EGP, SI variability, and the 10-90% glucose distribution remain physiologically plausible. The histogram aggregates all the 500 24h-long samples when generating new intraday parameters. It can be seen that by applying diurnal variability alone does not alter the distribution and matches the real-world distribution of CGM measurements. Sampling of new insulin sensitivity and endogenous glucose production values leads to a small portion of out-of-distribution samples, some of which may not be physiological.

Table 7 gives a more detailed picture of glucose metrics when new samples are generated for different patients. Overall, both the mean and the metrics that describe the tails of the distributions (TBR and TAR) remain close to the CGM measurements. There is a tendency for thicker tails as uncertainty increases from intraday to intra- and interday variability, but it is not true for all patients. For example, Patients 7 and 9 have marginally lower TBR and TAR values with both variabilities. Increasing the level of uncertainty also has a tendency to shift the mean of the distribution toward higher glucose values.

**Table 7:** Posterior predictive checks for all the participants based on glucose levels.

Patient	Mean BG			TIR			TBR			TAR		
	CGM	Intra	Inter	CGM	Intra	Inter	CGM	Intra	Inter	CGM	Intra	Inter
1	**100.1**	100.9	104.1	**91.8**	90.5	89.0	**8.2**	9.3	10.6	**0.0**	0.3	0.5
2	**141.4**	142.8	150.0	**80.1**	81.0	73.1	**2.4**	1.7	1.6	**17.5**	17.4	25.3
3	**164.3**	166.4	175.4	**63.7**	65.9	53.3	**0.3**	0.1	2.5	**36.0**	34.0	44.1
4	**141.6**	142.5	147.5	**80.2**	83.0	76.4	**3.5**	1.4	2.0	**16.2**	15.6	21.5
5	**119.1**	119.6	123.4	**88.0**	89.2	87.4	**6.2**	4.7	5.8	**5.9**	6.1	6.8
6	**141.1**	141.7	146.3	**75.1**	77.3	73.7	**6.5**	3.3	3.1	**18.4**	19.3	23.2
7	**149.3**	148.9	154.4	**70.7**	73.4	72.0	**1.0**	0.7	0.5	**28.3**	25.9	27.4
8	**181.0**	182.5	209.4	**48.2**	49.6	38.1	**0.8**	0.4	0.9	**51.0**	50.0	61.0
9	**148.4**	149.1	151.0	**73.4**	79.9	75.8	**3.2**	0.5	1.6	**23.4**	19.6	22.5
10	**155.0**	154.6	161.5	**60.8**	68.1	59.8	**4.4**	1.8	4.0	**34.8**	30.1	36.2

TIR: Time in range (%), TBR: Time below range (%), TAR: Time above range (%), Intra: Intraday variability is generated by sampling *SI*_*A*_ and $SI_{\Phi }$, Inter: Both intraday and interday variability is generated by sampling *SI*_*A*_, $SI_{\Phi }$, *SI*_*E*_, and *EGP*.

We evaluated the blood glucose prediction accuracy in the next hour as an additional means of validation. Prediction accuracy depends on the assumptions we make about the parameters of the disturbances. In particular, estimating these parameters requires time for their effects to develop. Thus we evaluated three scenarios: 1) The effect of meal, insulin, and physical activity in the last 45, 120, and 1440 minutes, respectively, are unknown. 2) The effect of meal, insulin, and physical activity in the last 30, 45, and 45 minutes, respectively, are unknown. 3) No disturbances are present in the last 45, 120, and 1440 minutes, respectively. These thresholds were necessary as the method is not for online blood glucose prediction. For EGP and SI, samples were drawn from the patient level posteriors. For scenario 1, the RMSE was 22.2 mg/dL at 30 minutes and 43.2 mg/dL at 60 minutes. For scenario 2, the RMSE was 17.0 mg/dL at 30 minutes and 33.9 mg/dL at 60 minutes. For scenario 3, the RMSE was 14.0 mg/dL at 30 minutes and 23.8 mg/dL at 60 minutes.

## Discussion

The low RMSE values and the relatively narrow credible intervals show that the SVI was able to consistently fit the model to the data. Largest discrepancies can be observed after the postprandial period, where measured glucose level can be lower. In future work, this dynamics might be addressed by differentiating glucose in the plasma and in the nonaccessible compartments such as adipose and muscle tissue. Due to the free-living nature of the data collection, the presence of unreported events is inevitable. However, we put emphasis on filtering the data to have more reliable sections and overall we observed good study compliance. Remaining missed events could also lead to larger differences in the predicted and measured trajectories, but can also contribute to misrepresentative posterior distributions.

Blood glucose prediction was not a primary focus of this work, however, we think it holds value as additional means for validation of the model. Blood glucose prediction in type 1 diabetes has a very wide literature with extremely diverse methods, datasets, and limited reproducibility. We compared reported prediction accuracies for 30 and 60 minutes of prediction horizons as it is the metric which is most often reported. Typically, reported RMSE values are between 16 and 27 mg/dL for the 30 minutes horizon, and between 28 and 35 mg/dL for the 60 minutes horizon [30–32]. Scenario 1 lies slightly above the reported range, but Scenario 3 lies below. This is due to the fact that Scenario 1 makes the assumption that, despite partially observing the effects of the disturbances, the method does not take them into account. Scenario 2 takes into account the total effect when 30-45 minutes have passed since the disturbance, and the results lie in the reported ranges. In Scenario 3, we filtered the samples when there is no disturbance present, which makes this setting ideal for observing how slowly changing trends in physiology are captured. In this setting, the achieved RMSE values are below the reported ones, which is expected, as disturbances cause the majority of fluctuations in blood glucose levels.

Posteriors for the phase of the sinusoidal intraday insulin variability showed wide credible intervals. To quantify possible patterns in intraday variability, we calculated the occurrences of peak insulin sensitivity for breakfast, lunch, and dinner similarly to the method in [22] with a notable difference. Instead of insulin sensitivity values, we focused on the distribution of the maximal insulin values between the three daily periods. It has been observed that diurnal insulin sensitivity peaks in the morning for healthy individuals – in type 1 diabetes the picture is not clear. Hinshaw et al. [33] reported large variability and opposite observation for the diurnal insulin sensitivity in type 1 diabetes compared to healthy ones. Hegab et al. [34] also observed lowered insulin sensitivity in the morning, but the study focused on adolescents whose metabolism is also affected by growth hormone which peaks during the morning and has anti-insulin effect. Lindmeyer et al. [35] focused on the “dawn” phenomenon and also observed large intraindividual variability in diurnal insulin requirements from patients with no apparent trends to patients with clear, increased insulin requirements at dawn. Our results are in line with previous studies, showing high variability, for 9 out of 10 patients insulin sensitivity was lowest during breakfast. Notable limitation is that a uniform prior was used only for the phase of the sinusoid in the model. Thus, it is easy to introduce residual disturbances and unmodeled phenomena into the sinusoidal intraday insulin sensitivity. For example, a larger glucose excursion that could not have been explained by self-reported events will most easily bleed into the sinusoid, even if it is not physiological variability but an unreported or unmodeled event. In future work, detection and estimation of missed meals could be taken into account.

Physical activity was found to be very important in explaining changes in insulin sensitivity for an extended period of time, with cases up to 48 hours. Not only the duration but also the effect on glycemia is substantial as well; without it, given the assumptions of the model, the mean glucose increase is predicted to be 18 mg/dL. The current model greatly abstracts away the underlying physiological effects of the physical activity compared to more nuanced methods [17,36]. This simplification was made intentionally as the methods in the literature are diverse and we aimed to capture the common aspects of them. In the future, a more detailed model of physical activity can be corporated. The posterior distributions of the parameters related to the physical activity show great variability which imply that the effect of physical activity has to be personalized and estimated at the event level.

In Fig 7 the generation of new daily samples for the patients diurnal variability leads to glucose traces that remain in the physiological 50-400 mg/dL range. When generating new insulin sensitivity and endogenous glucose production values, some out-of-distribution glucose values can be observed. The fitted model does not capture covariance between model parameters and inputs, and also potential nonlinear association between insulin sensitivity and endogenous glucose production. There is also a significant variance in the basal insulin which might explain some of the out-of-distribution glucose traces. These unmodeled phenomena could lead to out-of-distribution values. Overall, the high proportion of valid glucose traces implies that the SI, EGP, and intraday SI variability are fitted in a realistic manner, and can be used to generate stochastic glucose dynamics.

Roversi et al. [37] investigated meal carbohydrate counting accuracy among people with type 1 diabetes. For larger meals, they observed underestimation of the carbohydrate content compared to the values determined by dietitians. We did not observe such a tendency, in fact, based on the coefficient of the carbohydrate content in Table 6, larger meals were overestimated. In our study, there is no reference provided by dietitians and estimated carbohydrates are influenced by the assumptions of the model, fitting process, and the reported events. As the investigation of the the effect of the macronutritional composition was not the main focus of the study, we used a simplistic linear model for the post-hoc analysis, the model explains only little in the observed variability. Similarly to physical activity, the effect of the meal also show great variability from one meal to an other which again show the need to estimate the effects at the event level.

Interpretation of the results should be done by taking into account the main limitations of the study setup: 1) The investigated cohort is relatively well controlled, as the histogram in Fig 7 shows. 2) All the participants were on MDI therapy, but the method can be modified and extended to handle continuous insulin infusion. In fact, the underlying methodology simplifies as there is no distinction between the time constants and the timing, amount of insulin administration is more reliable. 3) SVI with a low-rank multivariate normal guide leads to an approximation of the true posterior unlike using Markov Chain Monte Carlo sampling which could (under sufficient conditions) converge to the true one. However, we believe that for the given task, which involves a high number of latent variables and high computational cost, SVI is better suited and makes the problem scalable for future work beyond 10 patients and 500 sections; the memory and computational limitations were noted in [8] previously as well.

The proposed method can be applied in several ways. It can serve as a replay methodology for patients and physicians, enabling the simulation of alternative hypotheses. The framework supports not only the simulation of a single trajectory, but also the representation of uncertainty bands arising from intra- and interday variability. Furthermore, its stochastic nature can be integrated into a closed-loop blood glucose control framework, enabling prediction under uncertainty. This would be an important feature, as overfitting insulin doses based on point estimates can cause difficulties in maintaining tight glucose ranges under various scenarios.

## Conclusion

We developed and investigated a probabilistic model of blood glucose dynamics that is able to represent inter- and intraday variability and takes into account the effect of physical activity. The method scaled well for multiple numbers of patients and multiple 24h-long sequences by utilizing SVI. We showed that the method was able to achieve a good fit across the whole cohort and represent inter-, intraday variability. The generation of new samples can be used to simulate stochastic virtual patient behavior and can be a suitable option for digital twin applications.

## Supporting information

S1 File Mathematical model. Detailed description of the deterministic submodel.
(PDF)
