# Supplementary material for: Stochastic virtual population in type 1 diabetes
Source: PLoS One. 2026 Feb 6;21(2):e0341034. doi: 10.1371/journal.pone.0341034 (PMC12880668; doi:10.1371/journal.pone.0341034)
Supplement: S1 File — Detailed description of the deterministic submodel. (PDF) [file pone.0341034.s001.pdf]

S1 File. Mathematical model.

Mate Siket, Gyorgy Eigner, Levente Kovacs

December 2025

1 Deterministic submodel

The glucose submodel is given as:

$$
\begin{aligned}
\dot{SG} &= (G - SG)\frac{1}{\tau_G}, \\
\dot{G} &= -(GEZI + h \cdot X)G + EGP \\
&\quad - SE \cdot E + R_A - F_R, \\
h &= \begin{cases} H, & \text{if } G < G_{th}, \\ 0, & \text{otherwise.} \end{cases} \\
F_R &= \begin{cases} 0.003(G - 162), & \text{if } G > 162 \text{ mg/dL}, \\ 0, & \text{otherwise.} \end{cases}
\end{aligned}
$$

The insulin submodel is given as:

$$
\begin{aligned}
SI &= SI_E \cdot (1 + P_A) \cdot (1 + SI_A \cdot \sin(2\pi(t + SI_\phi))), \\
\dot{X} &= -\frac{1}{\tau_{I4}} X + \frac{SI}{\tau_{I3}} I_p \\
\dot{I}_p &= -\frac{1}{\tau_{I3}} I_p + \frac{1}{\tau_{I2}} S_2 \\
\dot{S}_2 &= -\frac{1}{\tau_{I2}} S_2 + \frac{1}{\tau_{I1}} S_1 \\
\dot{S}_1 &= -\frac{1}{\tau_{I1}} S_1 + i
\end{aligned}
$$

The meal submodel is given as:

$$
\begin{aligned}
\dot{R}_A &= -\frac{1}{\tau_{D2}} R_A + \frac{1}{\tau_{D2}} D_2 \\
\dot{D}_2 &= -\frac{1}{\tau_{D2}} D_2 + \frac{1}{\tau_{D1}} D_1 \\
\dot{D}_1 &= -\frac{1}{\tau_{D1}} D_1 + \frac{1}{V_G} m
\end{aligned}
$$

The physical activity submodel is given as:

$$\begin{aligned}
P_A &= P_{A,long} + P_{A,short}, \\
\dot{P}_{A,long} &= -\frac{1}{\tau_{P,long}} P_{A,long} + P_{long} pa, \\
\dot{P}_{A,short} &= -\frac{1}{\tau_{P,short}} P_{A,short} + P_{short} pa, \\
\dot{E} &= -\frac{1}{\tau_E} E + \frac{1}{\tau_E} E_1, \\
\dot{E}_1 &= -\frac{1}{\tau_E} + hr.
\end{aligned}$$

SG is the subcutaneous glucose concentration, G is the blood glucose concentration, h is the coefficient for insulin sensitivity in hypoglycemia, F_R is the renal excretion, SI is the varying insulin sensitivity, X is the effect of insulin, R_A is the rate of appearance of glucose from meal intakes, P_A is the effect of physical activity on insulin sensitivity, E is the energy expenditure with respect to elevated heart rate.

Node	Variable	Level	Description
M	T_D	Meal	Meal start offset. Uncertainty in the self-reported time, delay in absorption
M	τ_{D1}	Patient	Meal absorption time constant 1
M	τ_{D2}	Patient, Meal	Meal absorption time constant 2.
M	M_A	Meal	Carb coefficient. Uncertainty in the self-reported carbohydrate content.
V_G	V_G	Cohort, Patient	Glucose distribution volume.
m	Self-reported carb content	Meal (Observed)	
I	T_I	Bolus	Bolus start offset. Uncertainty in the self-reported time, delay in absorption.
I	τ_{I1}	Patient, Bolus	Bolus time constant 1. Captures variability in absoprtion with respect to injection site.
I	I_A	Bolus	Bolus coefficient. Uncertainty in the self-reported bolus injection.
i	Self-reported bolus	Meal (Observed)	Short-acting insulin
X_0	X_0, S_0, I_{p0}	Cohort, Patient, Sample	Initial state of the model, glucose values are based on CGM at 0 time instance.

EGP	EGP	Sample	Endogenous glucose production: $\mathcal{TN}(EGP_i + EGP_m \cdot SI_E, \sigma)$
EGP	EGP_i	Cohort, Patient	Endogenous glucose production at zero insulin sensitivity
EGP	EGP_m	Patient	Slope between SI and EGP
C	$GEZI$	Cohort, Patient	Glucose effectiveness at zero insulin $GEZI$
C	$\tau_{I2,3,4}$	Cohort, Patient	Insulin absorption time constants
C	τ_G	Cohort, Patient	Delay due to subcutaneous measuring site of the glucose.
C	H	Cohort, Patient	Effect of increased insulin sensitivity in hypoglycemia.
C	G_{th}	Cohort, Patient	Threshold glucose level for the increased insulin sensitivity in hypoglycemia.
C	SE	Cohort, Patient	Effect of elevated metabolic rate parameterized by excess heart rate over the resting value.
C	τ_E	Cohort, Patient	Time constant of the metabolic rate.
SI	SI_E	Cohort, Patient, Sample	Expected value of the insulin sensitivity without physical activity and intraday variation.
SI	SI_A	Cohort, Patient, Sample	Amplitude of the intraday sinusoidal insulin sensitivity variation.
SI	SI_Φ	Cohort, Patient, Sample	Phase of the intraday sinusoidal insulin sensitivity variation.
P_A	T_P	Exercise	Uncertainty in the self-reported time
P_A	$\tau_{P,long}$, $\tau_{P,short}$	Patient, Exercise	Time constant of the short and long effect of physical activity.
P_A	P_{long}, P_{short}	Exercise	Gain of the short and long effect of physical activity.
p	Self-reported physical activity	Exercise (Observed)	
hr	Measured heart rate	Measurement (Observed)	
CGM	Measured blood glucose	Measurement (Observed)	

SG	Subcutaneous glucose level	Variable (Observed)	Model output

Table 1: **Summary for the latent and observed variables of the model.**
